# Sensitivity analysis of RT-qPCR and RT-ddPCR for SARS-CoV-2 detection with mutations on N1 and E primer-probe region

**DOI:** 10.1128/spectrum.04292-23

**Published:** 2024-06-25

**Authors:** Shiyou Liu, Xiaoru Chai, Chao Liu, Jiaxuan Bai, Juntao Meng, Hong Tian, Xu Han, Guangyue Han, Qi Li, Xiangdong Xu

**Affiliations:** 1 Hebei Key Laboratory of Pathogens and Epidemiology of Infectious Diseases, Hebei Provincial Center for Disease Control and Prevention, Shijiazhuang, China; 2 School of Public Health, Hebei Medical University, Shijiazhuang, China; 3 Shijiazhuang Qiaodong Sewage Treatment Plant, Shijiazhuang, China; 4 Hebei Key Laboratory of Environment and Human Health, Shijiazhuang, China; Shandong First Medical University, Jinan, Shandong, China

**Keywords:** SARS-CoV-2, mutation, RT-qPCR, RT-ddPCR, primer-probe region

## Abstract

Severe acute respiratory syndrome coronavirus 2 (SARS-CoV-2) is an RNA virus that undergoes rapid mutation. Based on viral whole genome sequencing analysis in Hebei Province, China, we identified several essential single nucleotide variants (SNVs) on primer-probe regions accumulating within some Omicron variants’ genomes. In this study, we focused on three SNVs, C28290T, T28297C, and C28311T emerging on 2019-nCoV-N1 (CDC-N1) primer-probe regions, recommended by CDC in 2020, and two SNVs, C26270T, A26275G emerging on E (Charité-E) primer-probe regions recommended by Charité, Germany. Our findings revealed that the presence of one or two SNVs in the primer or probe region affected the sensitivity of reverse transcription-quantitative polymerase chain reaction and droplet digital PCR to varying extents. This discovery underscores the importance of continuously monitoring the whole genome sequences of SARS-CoV-2 variants, especially the primer-probe targeting regions, and correspondingly updating commercial test kits or recommended primer-probe sequence sets.

**IMPORTANCE:**

The emergence of new severe acute respiratory syndrome coronavirus 2 (SARS-CoV-2) variants has resulted in a growing number of mutations in its genome, presenting new challenges for the diagnosis of SARS-CoV-2 using reverse transcription-quantitative polymerase chain reaction (RT-qPCR) and droplet digital PCR (RT-ddPCR) methods. There is an urgent need to develop refined methods for modifying primers and probes to improve the detection of these emerging variants. In this study, our focus was on the SNVs that have emerged in the CDC-N1 and Charité-E primer-probe regions. Our research has confirmed that the presence of these SNVs in the primer or probe region can significantly affect the results of coronavirus disease 2019 tests. we have developed and validated a modified detection method that can provide higher sensitivity and specificity. This study emphasizes the importance of refining the primer-probe sets to ensure the diagnostic accuracy of RT-qPCR and RT-ddPCR detection.

## INTRODUCTION

Monitoring and identifying prevalent severe acute respiratory syndrome coronavirus 2 (SARS-CoV-2) variants and timely updating detection methods are crucial for diagnosing COVID-19 (coronavirus disease 2019) infection (1). Genome sequencing should include all primer and probe targeting regions to ensure reliable detection of SARS-CoV-2 (2). The Ct value or RNA copies serve as critical laboratory indicators for determining hospitalization in COVID-19 patients and providing essential information for public health policy. Therefore, it is very important to report the correct Ct value and copies of SARS-CoV-2 RNA for guiding clinical practice and infection control (3–5).

Greater attention should be paid to the high variability of SARS-CoV-2 to avoid false negative detection results. A recent study by Leuzinger et al. discovered that the globally spreading Omicron BA.2 and BA.5 variants significantly reduced the positive detection rates of widely used antigen tests, possibly due to variations in the nucleocapsid protein (N) (6). Considering that mutations in SARS-CoV-2 might compromise assay accuracy, this could result in inconsistent performance and increase the risk of false negatives (7). Therefore, we have focused on assessing mutations in the primer-probe sequences and their potential impact.

In this study, we employed CDC-N1 and Charité-E primer-probe sets, as well as customized mutated primer-probe sets, to detect both mutated and non-mutated samples simultaneously. Additionally, we assessed whether sensitivity was affected.

## MATERIALS AND METHODS

### Clinical samples

Starting from 8 January 2023, a continuous program was implemented in Hebei Province, China, to track the variants of SARS-CoV-2. The viral whole genome sequences were obtained from oral samples collected from sentinel hospitals, which tested positive for SARS-CoV-2 nucleic acid. Among these samples, three exhibited a single nucleotide variant (SNV) (T28297C) in the CDC-N1 forward primer region, two had two SNVs (C28290T and T28297C) in the CDC-N1 forward primer region, and one sample had no mutations. These six samples were numbered as follows: HS-139, DZ-77, SJZ-447, XJ-135, DZ-76, and DZ-79. All six samples exhibited the presence of an SNV (C28311T) within the CDC-N1 probe region, as well as two SNVs (C26270T and A26275G) in the Charité-E forward primer region.

### Primer-probe sets

**Fig 1:**
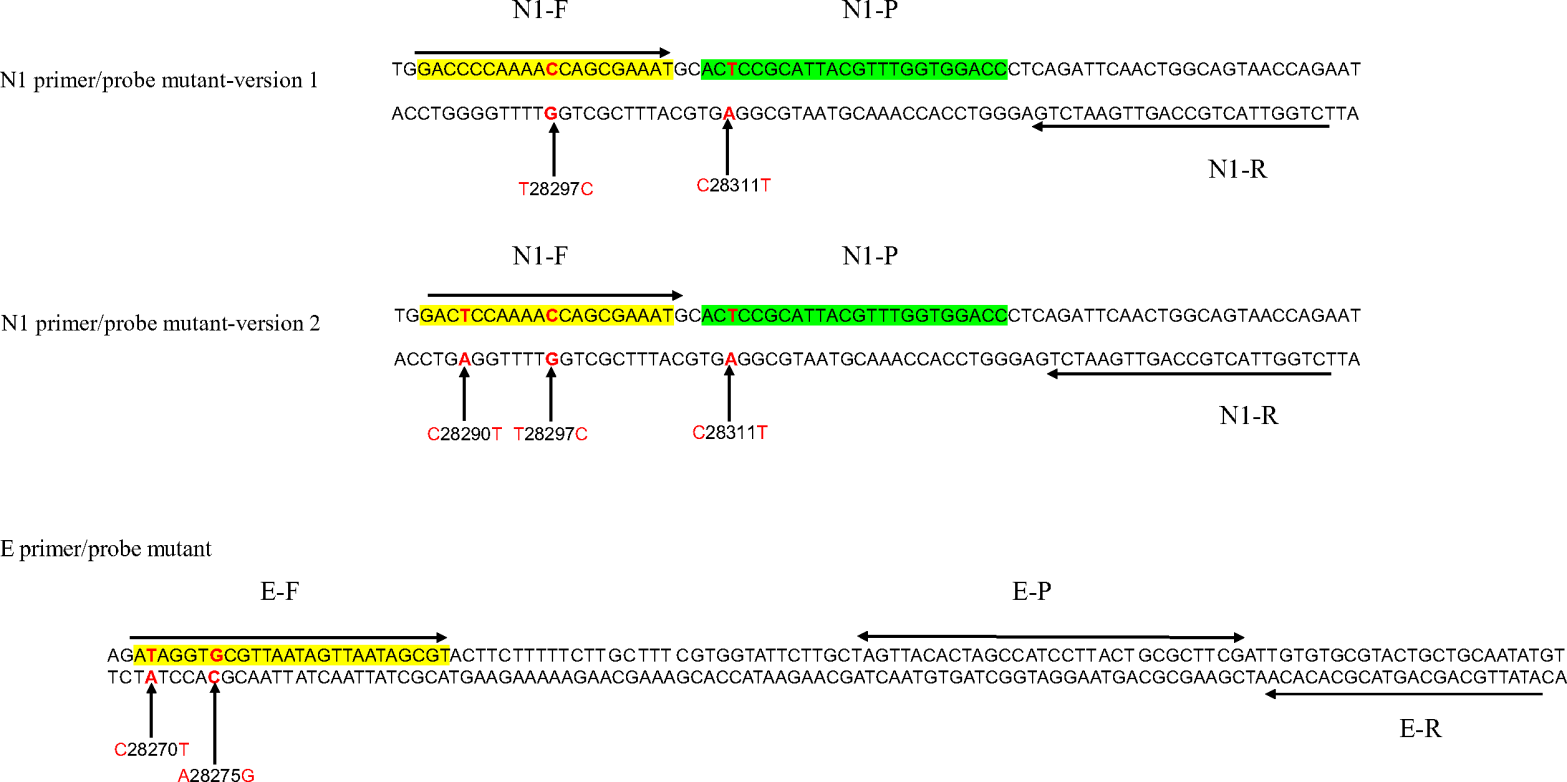
N1 mutant 28285-28360 sequence: N1 primer/probe mutant version 1, N1 primer/probe mutant version 2 and E mutant 26267-26383 sequence: primer/probe mutant.

The original CDC-N1 primer-probe set (8), Charité-E primer-probe set (9), and their mutant sets (Fig. 1) were synthesized by a commercial company (Generay Biotech, Shanghai, China). The probes for CDC-N1 and Charité-E were labeled with Fluorescein Amidite(FAM) and Violet Invader(VIC) fluorophore, respectively. All quenching groups were designed as DUQ dual quenching probes.

### RT-qPCR and RT-ddPCR

The reverse transcription-quantitative polymerase chain reaction (RT-qPCR) assay was performed following the manufacturer’s instructions with the AgPath-ID One-Step RT-PCR Reagents Kit (item no. AM1005, Thermo Fisher Scientific, Massachusetts, USA) and analyzed using QuantStudio 7 Flex. The Ct value level was automatically determined by the software, and the positive samples should exhibit sigmoidal curves.

Reverse transcription-droplet digital PCR (RT-ddPCR) detection was performed using the One-step RT-ddPCR Advanced Kit for Probes (Bio-Rad, California, USA). The RT-ddPCR system and procedure were optimized for different primer-probe combinations to maximize the number of positive droplets and ensure clear separation between positive (blue) and negative (gray) droplets. Subsequently, the entire 20 µL of each reaction mixture was emulsified into nanoliter droplets using 70 µL of droplet generator oil (Bio-Rad, Hercules, CA, USA) performed on a QX200 droplet generator (Bio-Rad, Hercules, CA, USA), and then sealed with a PX1 thermal sealer (PX1 PCR Plate Sealer). The sealed 96-well PCR plate was placed in the C1000 Thermal Cycler for PCR reaction (C1000 Touch Thermal Cycler). After thermal cycling, the 96-well PCR plate was transferred to the QX200 Droplet Reader to read the droplets. The target amplification products were quantified using QuantaSoft software after reading. The reaction systems and procedures of RT-qPCR and RT-ddPCR are detailed in Table 1.

**TABLE 1:** RT-qPCR and RT-ddPCR reaction mix preparation and thermal cycling condition

RT-qPCR				
25 µL RT-qPCR reaction	Volume per reaction, μL			
2X RT-PCR buffer	12.5			
N1/E				
Forward primer (*c* = 10 µmol/L)	0.5/0.5			
Reverse primer (*c* = 10 µmol/L)	0.5/0.5			
Probe (*c* = 5 µmol/L)	0.3/0.3			
25X RT-PCR enzyme mix	1			
RNA	4			
Nuclease-free water	4.9			

Step	Temperature, °C	Time	Cycles	
Reverse transcription	45	10 min	1	
RT inactivation/initial denaturation	95	10 min	1	
Amplification	95	15 s	40	
	60	45 s	40	

RT-ddPCR for mutations				
20 µL ddPCR reaction	Volume per reaction, μL			
Supermix (one-step RT-dd PCR)	5			
Reverse transcriptase	2			
300 mM DTT	1			
N1/E				
Forward primers (*c* = 10 µmol/L)	0.9/0.9			
Reverse primers (*c* = 10 µmol/L)	0.9/0.9			
Probes (*c* = 5 µmol/L)	0.5/0.5			
Total RNA	4			
RNase-/DNase-free water	3.4			

Cycling step	Temperature, °C	Time	Number of cycles	Cycling step
Reverse transcription	45	60 min	2°C/s for all	1
Enzyme activation	95	10 min		1
Denaturation	94	30 s		40
Annealing/extension	57.3	120 s		40
Enzyme deactivation	98	10 min		1
Hold	4	Infinite		1

RT-ddPCR for primeval				
20 µL ddPCR reaction	Volume per reaction, μL			
Supermix (one-step RT-dd PCR)	5			
Reverse transcriptase	2			
300 mM DTT	1			
N1/E				
Forward primers (*c* = 10 µmol/L)	0.9/0.9			
Reverse primers (*c* = 10 µmol/L)	0.9/0.9			
Probes (*c* = 10 µmol/L)	0.5/0.5			
Total RNA	4			
RNase-/DNase-free water	3.4			

Cycling step	Temperature, °C	Time	Number of cycles	Cycling step
Reverse transcription	45	60 min	2°C/s for all	1
Enzyme activation	95	10 min		1
Denaturation	94	30 s		40
Annealing/extension	60.2	90 s		40
Enzyme deactivation	98	10 min		1
Hold	4	Infinite		1

## RESULTS AND DISCUSSION

Research tracking SARS-CoV-2 variations in Hebei Province, China, has revealed that, alongside the SNV C28290T, four additional SNVs—T28297C, C28311T, C26270T, and A26275G—were present in almost all samples. Genomic analysis of SARS-CoV-2 using the Nextstrain platform (10) revealed that the normalized Shannon entropy of the SNV (C28290T) is 0.016, and it is predominantly present in XBB.1.16, EG.5.1, and their respective sub-branches. This particular SNV was initially discovered in May 2023 and is currently only occurring in China. We regard this inherited variation as noteworthy. The SNV (T28297C) emerged in September 2022, mainly within XBB.1.16 and XBB.1.9 sub-branches, with a normalized Shannon entropy of 0.684. The SNVs (C28311T and C26270T) earliest emerged in 2020 and were inherited by almost all Omicron variants and currently exhibit a trend of reversion mutations in India and New Zealand with respective normalized Shannon entropies of 0.402 and 0.406. The SNV (A26275G) mutation took place in BA.2.75, XBB, and its sub-branches in December 2021 with a normalized Shannon entropy of 0.562.

**Fig 2:**
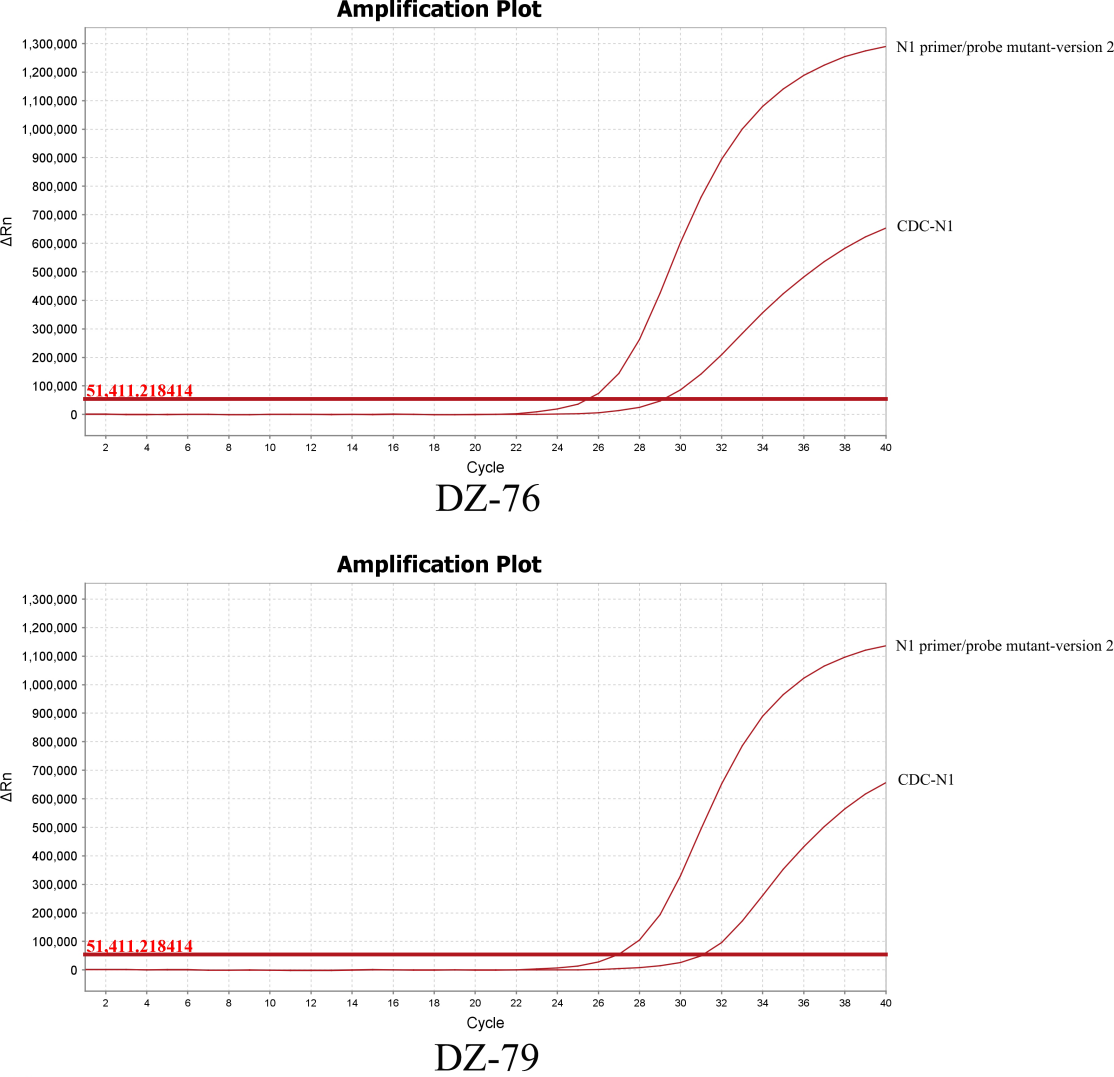
When the three SNVs C28290T, T28297C, and C28311T occur in the DZ-76 and DZ-79 N1 genes of the samples to be tested, the CDC-N1 primer-probe set and the N1 primer/probe mutant version 2 are used to detect the N1 gene in the samples, respectively, to obtain the Ct value. The difference in Ct values was found to be approximately 3.84 ± 0.20 $\left(\bar{x}\pm s\right)$ with lower values measured using N1 primer/probe mutant version 2.

In this study, we collected six oral samples in total and primarily focused on five mutations present in the N and E primer-probe region, which may change the sensitivity of the PCR test. Through RT-qPCR, we observed that the Ct value using the CDC-N1 original primer-probe sets is approximately 0.40 ± 0.17 $\left(\bar{x}\pm s\right)$ higher than that using their mutant sets when one SNV (T28297C) is present in the N1 forward primer region and one SNV (C28311T) is present in the N1 probe region. In RT-ddPCR, we observed that the concentration of N1 copies using CDC-N1 primer-probe sets were 1.31 ± 0.36 $\left(\bar{x}\pm s\right)$ times lower than using their mutant sets. Detailed data of RT-qPCR and RT-ddPCR for all samples are shown in Table 2. However, when two SNVs (C28290T and T28297C) are present in the CDC-N1 forward primer region and one SNV (C28311T) is present in the probe region, the Ct value was 3.84 ± 0.20 $\left(\bar{x}\pm s\right)$ higher than that using its mutant sets (Fig. 2). From a molecular biology standpoint, we can perform a comprehensive analysis of this phenomenon. SARS-CoV-2 has a genome consisting of multiple genetic regions. Among these, the N1 gene holds a pivotal role and is often used as a key target for detection. Primers and probes are critical for specific binding to SARS-CoV-2, which is essential for guiding accurate PCR amplification. However, we have identified three SNVs within the N1 gene primer-probe region. These mutations could reduce the binding affinity of the original primer-probe sets to the virus. A lower match between the primer-probe and their target sequences severely affects PCR amplification efficiency, thus undermining the sensitivity and precision of RT-qPCR assays. Furthermore, we observe that more SNVs in this region markedly degrade diagnostic accuracy.

**TABLE 2:** Test results of RT-qPCR and RT-ddPCR for all samples

Name	SNVs	Sample	Primer/probe mutant version				CDC-N1 and Charité-E sets			
			RT-qPCR (Ct)		RT-ddPCR (copies/μL)		RT-qPCR (Ct)		RT-ddPCR (copies/μL)	
			N1	E	N1	E	N1	E	N1	E
N1-F	T28297C	HS-139	20.751	18.957	10,400	9,000	20.945	18.996	10,000	504
		HS-139	20.716	18.941	9,000	7,900	21.200	19.578	9,800	455
		DZ-77	27.460	27.844	96	15.5	27.996	28.733	51.5	1.9
		DZ-77	27.754	27.937	86	13.6	27.949	28.420	56	1.5
		SJZ-447	30.732	29.366	10.8	5.5	31.143	30.710	7.6	0.6
		SJZ-447	30.698	29.677	10.4	5.2	31.288	30.345	9.5	0.6
	None	XJ-135	30.858	30.055	5.2	2.2	30.922	31.265	5.2	0.3
		XJ-135	31.030	30.340	3.3	2.7	30.685	31.283	4.0	0.42
	C28290T, T28297C	DZ-76	25.511	24.175	518	539	29.083	24.425	13.4	56
		DZ-76	25.443	24.291	508	535	29.297	24.514	12.6	50.2
		DZ-79	27.120	28.143	147	31.2	30.983	28.681	6.9	7.8
		DZ-79	26.919	28.040	144	31.2	30.975	28.731	7	7.7

**Fig 3:**
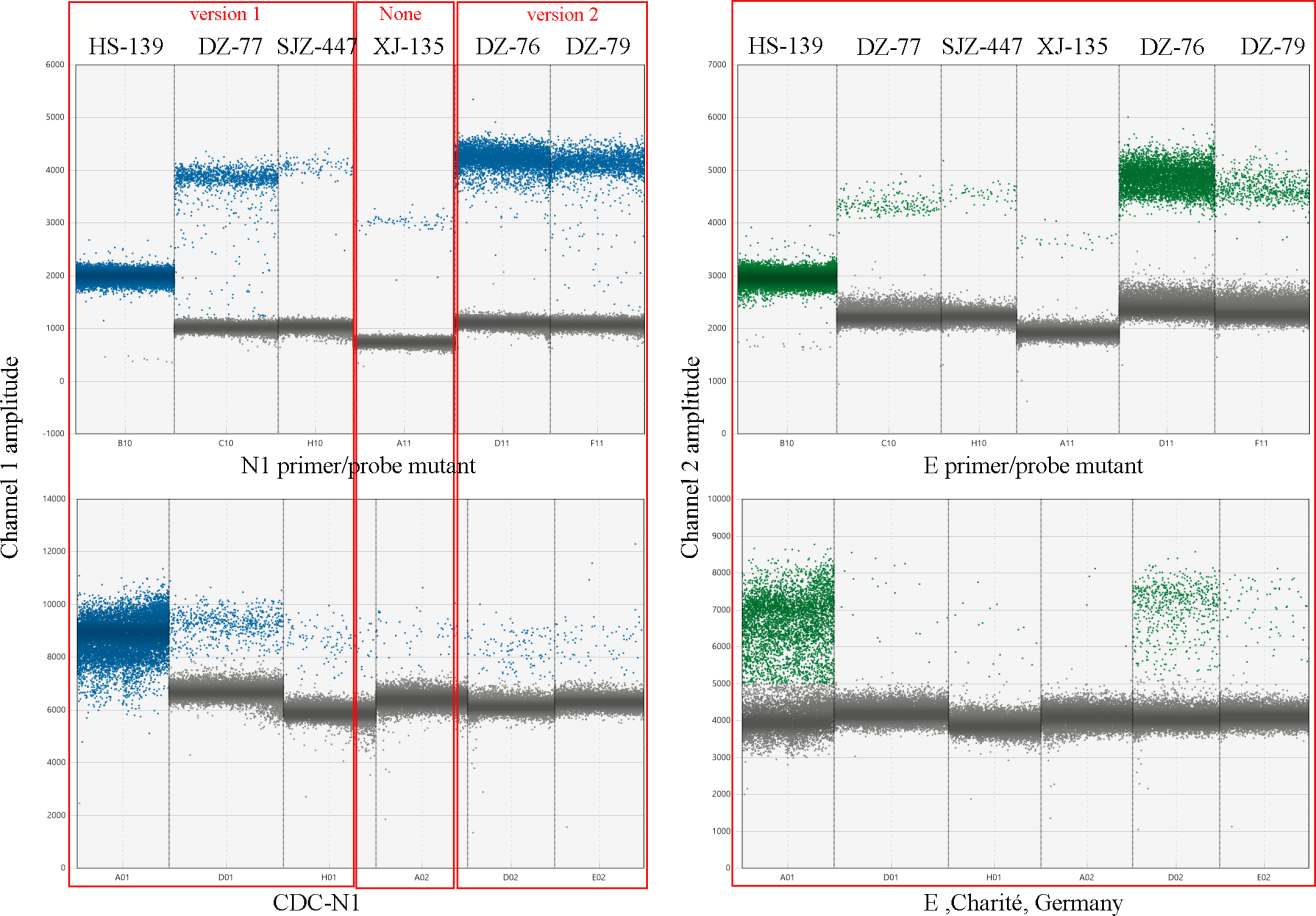
Samples were detected by RT-ddPCR using original primer-probe sets and primer-probe mutant sets, respectively.The blue droplet represents the N1 gene and the green droplet represents the E gene.

By RT-ddPCR, the concentration of N1 copies was 30.21 ± 10.73 $\left(\bar{x}\pm s\right)$ times lower when the CDC-N1 primer-probe set was used compared with its mutant set. The 2D illustration of RT-ddPCR detection is shown in Fig. 3. The results show that if the CDC-N1 primer-probe set was used, especially for low-concentration mutation samples with two SNVs (C28290T and T28297C) in the forward primer region and one SNV (C28311T) in the probe region, false negative results may occur. Over an extended period, the viral load of SARS-CoV-2 in COVID-19 patients or in the environment may be underestimated by using the original CDC-N1 primer-probe set. When mutations (C26270T and A26275G) emerged on the Charité-E primer-probe regions of the E gene, simultaneous detection using RT-qPCR and RT-ddPCR showed a Ct value of 0.66 ± 0.39 $\left(\bar{x}\pm s\right)$ higher than that using its mutant set, with the copies being approximately 7.72 ± 2.27 $\left(\bar{x}\pm s\right)$ times lower. Additionally, these results demonstrate that RT-ddPCR may exhibit greater sensitivity to accurate primer-probe sequences compared to RT-qPCR. Inaccurate primer-probe design may have a greater impact on the detection results, leading to a higher likelihood of missed COVID-19 diagnoses.

The results indicate that using only the US CDC 2019-nCoV-N1 or Charité, Germany E primer-probe sets for RT-qPCR and RT-ddPCR testing of SARS-CoV-2 concentration in samples may result in the “false-negative” of COVID-19 patients with low viral loads. A previous study also reported the poor performances of UCDC-N1 and Charité-E sets in wastewater testing in Hong Kong (11). The study revealed that the limit of detection for these two sets exceeded 10 copies/μL. Additionally, a stealth SARS-CoV-2 Omicron variant was identified, which displayed insensitivity to RT-qPCR with N1 and N2 primers in Japan (12). Therefore, if only the RT-qPCR system using the N1 and N2 primer-probe sets is used to screen COVID-19 patients, some of them may be overlooked. Based on these findings, it is recommended that laboratories promptly develop a new primer-probe set when monitoring a mutant with long-term stable variation in a specific location. Moreover, diagnostic kits should be regularly updated to accommodate the emergence of new variants.

SARS-CoV-2, particularly its Omicron variant, has exhibited a notable decline in virulence and pathogenicity, yet its transmission capability remains formidable. Consequently, we cannot overlook any potential future alterations in SARS-CoV-2. If SARS-CoV-2 acquires an enhanced tropism for the lungs during its transmission, it may once again pose a severe threat to human health. This underscores the importance of continuously monitoring and addressing SARS-CoV-2. Mutations in the primer-probe regions can hinder the identification and tracking of virus variants. Therefore, it is crucial to promptly update and optimize the design of PCR detection reagents to ensure their effective binding with mutated virus sequences.

### Conclusion

The emergence of new SARS-CoV-2 variants poses new challenges for diagnosis due to the accumulation of mutations in the viral genome. Stable occurrences of SARS-CoV-2 variants have been observed, characterized by SNVs at the N1 forward primer region (T28297C), the N1 probe region (C28311T), and the E forward primer region (C26270T and A26275G). Our investigations have confirmed that the presence of one or two SNVs in the primer or probe regions can interfere with the sensitivity of both RT-qPCR and RT-ddPCR at varying degrees. This study emphasizes the importance of modifying primer/probe sequences to enhance diagnostic effectiveness.
